# Theoretical study of electronic structure, lone pair localization, and electronic transport properties of unconventional bulk and 2D γ-SnSe and γ-SnS†

**DOI:** 10.1039/d5ra01965f

**Published:** 2025-05-16

**Authors:** Nguyen Truong Long, Huynh Anh Huy, Neeraj Mishra, Guy Makov

**Affiliations:** a School of Physics Education, Can Tho University Can Tho 900000 Vietnam truonglong@ctu.edu.vn; b Dept. of Materials Engineering, Ben-Gurion University of the Negev Beer Sheva 84105 Israel; c Department of Chemical and Materials Engineering, University of Alberta Edmonton AB T6G2V4 Canada

## Abstract

Tin-based monochalcogenides, particularly SnSe and SnS, are of growing interest due to their cost-effectiveness, environmental compatibility, and exceptional thermoelectric properties. Beyond the conventional α-*Pnma* phase, these materials can adopt alternative bulk and low-dimensional structures with distinct electronic and transport characteristics. The recent experimental discovery of a layered γ-*Pnma* SnSe phase in 2023 has further stimulated the search for novel structural allotropes within this family and the assessment of their electronic properties. In this study, we employ density functional theory to examine the structural stability, electronic structure, lone-pair characteristics, and thermoelectric performance of γ-SnS and γ-SnSe in both bulk and two-dimensional (2D) monolayer forms. Our results demonstrate that γ-SnSe and γ-SnS monolayers are thermodynamically stable and can be synthesized *via* mechanical exfoliation. Electronic structure analysis reveals a substantial band gap expansion in the 2D monolayers, increasing by a factor of 4 to 20 compared to the bulk. A detailed investigation of localized lone pairs in the 2D monolayers identifies two distinct p-state contribution schemes for γ- and α-monolayers, with a notable involvement of the Sn 5p state. Additionally, both bulk and monolayer γ-SnSe and γ-SnS exhibit large Seebeck coefficients and power factors, comparable to or exceeding those of the conventional α-*Pnma* phases.

## Abstract 2

Evaluation of electronic structure, lone pairs, and potential thermoelectric transport properties of unconventional monolayer and bulk γ-*Pnma* SnS and SnSe.

## Graphical abstract

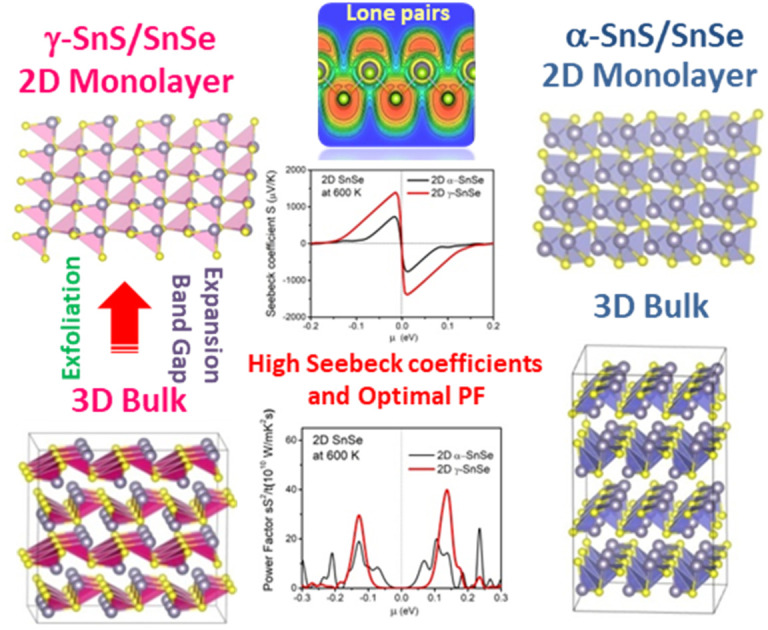

## 1 Introduction

In recent years, tin monochalcogenides, such as SnS and SnSe, have attracted significant research interest as functional materials for optoelectronic and thermoelectric applications.^1–4^ The discovery of an extraordinary thermoelectric figure of merit (*ZT* > 2) in conventional α-*Pnma* SnSe,^5^ together with the promising thermoelectric properties of related monochalcogenides, including SnS, SnTe, PbS, and PbTe (*ZT* of 1.05, 1.46, 1.51, and 1.59, respectively) makes them candidate thermoelectric materials.^6^ Notably, SnS and SnSe are economical, low toxic, and earth-abundant, so they are attractive functional materials for photovoltaic and thermoelectric applications in comparison with the current materials.^3^ Beyond their thermoelectric applications, SnS and SnSe compounds exhibit attractive electronic and optical properties for extensive applications in optoelectronic and photovoltaic devices.^7–11^

The conventional structure of SnSe and SnS is space group *Pnma* (*Z* = 62), denoted as α-phase, is a layered crystal and possesses an indirect bandgap of 0.9–1.0 eV.^12,13^ They have potential utility as an absorber material in solar cells due to its high absorption coefficient and high hole mobility.^7^ Especially, α-SnSe has excellent thermoelectric conversion ability, with a figure of merit of ∼2.6 at 923 K, due to its low thermal conductivity and high power factor.^11,14^ Therefore, SnSe crystals are currently being developed as an economical alternative with superior performance for commercial thermoelectric cooling, along with several high potential thermoelectric materials such as highly disordered or layered compounds Cu_2−*x*_S, Cu_2−*x*_Se, AgSbSe_2_, In_4_Se_3_, and CsAg_5_Te_3_ (ref. 15 and 16) to replace the present Bi_2_Te_3_-based alloy.^3,17^ Recently, two unconventional metastable phases of SnS and SnSe, namely layered γ-SnSe and cubic π-SnSe/SnS, have been discovered experimentally and possess semiconductor gaps varying from ultranarrow band gap of 0.095 eV in γ-*Pnma* SnSe^18,19^ to wider band gaps of 1.53/1.28 eV in π-*cubic* SnS/SnSe,^20,21^ respectively. These novel allotropes of SnSe and SnS exhibit a range of versatile properties, such as small electron effective mass, low thermal conductivity, and high optical absorption coefficients similar to conventional α-structure.^17^ Furthermore, the unconventional phases can enable band gap selection, exhibit anisotropy, and facilitate unique nanoscale morphologies such as nano-size particles or low-dimensional structures.

Since SnS and SnSe phases exhibit layered structures, it is possible to form two-dimensional (2D) monolayers experimentally by exfoliation or Li-intercalation.^22–25^ Among the 2D layered materials, 2D α-SnS and α-SnSe have been extensively explored for electronic structure, mechanical properties, and versatile applications in thermoelectric and optoelectronics^10,26–30^ In particular, remarkable thermoelectric properties have been reported for the conventional 2D α-monolayer SnS and SnSe relative to 3D bulk phase, demonstrating their high power factor and considerable *ZT* values.^2,29,30^ 2D monolayers of SnS and SnSe could maintain high stability, high power factor, and low lattice thermal conductivity.^30^ This leads to estimated *ZT* values of nearly 0.8 for 2D α-SnS and 2D α-SnSe at 900 K.^30^ Thus, 2D monolayers of SnS and SnSe are considered mid-temperature thermoelectric materials.^2^

Aside from the conventional α-structure SnS and SnSe in 2D and bulk phases, several novel unconventional 3D and 2D structures of SnSe have been discovered or predicted to expand the potential applications of this family in optoelectronic and energy conversion devices.^13,31–34^ It is interesting to examine the thermoelectric transport characteristics of unconventional bulk and 2D structures, such as the newfound γ-phase of SnSe and SnS, as they could possess similar or better properties than the conventional α-structure. In addition, exploring new structures helps develop our fundamental understanding of the electronic and transport characteristics of this family of thermoelectric functional materials, *e.g.* ref. 13. Particularly, the presence of stereochemically active lone pairs in SnS and SnSe induces the manifestation of multiple polymorphs from the symmetric rock salt structure. Therefore, the diversity of lone pair presence could be a major avenue to understanding the electronic structure and interatomic bonding in SnS and SnSe, which leads to enhanced thermoelectric properties. Motivated by the recent development of low-dimensional structures of SnS and SnSe, our study focuses on two-dimensional structures of γ-SnS and γ-SnSe based on the synthesized novel bulk phase.^19^ We explore the electronic and electronic transport properties of 2D γ-SnS and γ-SnSe to evaluate their potential application in thermoelectric devices. We also compare the electronic structures, presence of lone pair, and electronic transport properties between the 3D and 2D structures of γ-phase to provide an extensive evaluation of this unconventional structure.

## 2 Calculation methods

We performed DFT calculations with a plane wave basis set using the Quantum Espresso suite of software.^35^ Our DFT calculation employed ultrasoft pseudopotentials^36^ from PS library^37^ based on generalized gradient approximation (GGA) by the Perdew–Burke–Ernzerhof (PBE) exchange-correlation functional.^38^ In addition, we utilized the hybrid functionals PBE0(ref. 39) to improve the accuracy of electronic structure and included van der Waals (vdW) interactions based on nonlocal correlation functional vdW-DF3-opt1.^40,41^ The pseudopotentials accommodate fourteen valence electrons of Sn (4d^10^3s^2^3p^2^) and six valence electrons of S (3s^2^3p^4^) and Se (4s^2^4p^4^). To obtain the optimized structure of monolayers for both α- and γ-structures of SnS and SnSe, we used variable-cell relaxation conditions at a cutoff energy of 60 Ry to achieve reasonably converged energy and calculation cost. The convergence threshold for the self-consistent field calculations is at 10^−8^ eV, and the optimal force condition for variable-cell relaxation is 10^−4^ eV Å^−1^. Our calculations utilize Monkhorst–Pack *k*-grid^42^ of 8 × 8 × 8 *k*-points for 3D structure and 16 × 16 × 1 for 2D structure. For the 2D structure, we added a vacuum space of 20 Å to eliminate the effect of periodic images. The dynamical stability of our 2D monolayer phases of SnS and SnSe was evaluated by calculating the phonon dispersion curves using density functional perturbation theory^43^ with grids of 12 × 12 × 1 in *k*-space and a 3 × 3 × 1 *q*-mesh. The electronic structure and the projected density of states (PDOS) were calculated for the final optimized structures. Interpretation of crystal orbital Hamilton population (COHP)^44^ is applied to investigate the lone pairs in each monolayer structure. The energy range corresponded to lone pairs in the COHP analysis is used to calculate the integrated density of states (IDOS) for each contributor orbitals to the lone pairs. Electronic transport properties of 2D SnS and SnSe structures, including Seebeck coefficient and electrical conductivity, were calculated using semi-classical Boltzmann transport theory as developed in BoltzTraP2,^45^ widely applied by various DFT calculations for thermoelectric research. We employed BoltzTraP2 under two approximations of the rigid band and constant electron relaxation time, *τ*, (CRTA). Transport properties such as electrical conductivity (*σ*), Seebeck coefficient (*S*), and power factor (PF = *S*^2^*σ*) are determined as a function of the chemical potential, *μ*, and temperature, following other studies of thermoelectric materials.^46–48^ The Boltzmann's transport equation for electrical conductivity and Seebeck coefficient are described in BoltzTrap2(ref. 45) as follows:

Transport distribution function:1
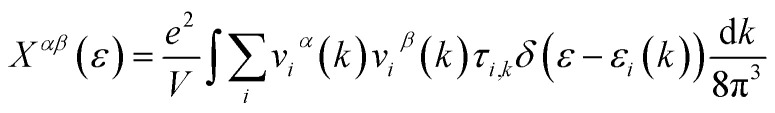


Electrical conductivity:2
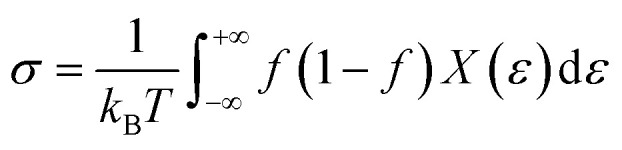


Seebeck coefficient:3
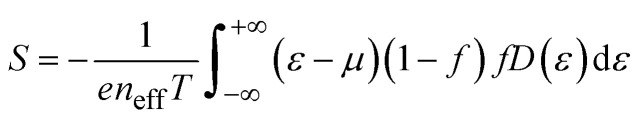
where the 
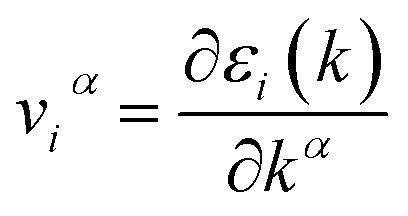
 is the group velocity, *τ*_*i*,*k*_ is the relaxation time, *f*(*ε*;*μ*,*T*) is the Fermi distribution function, 
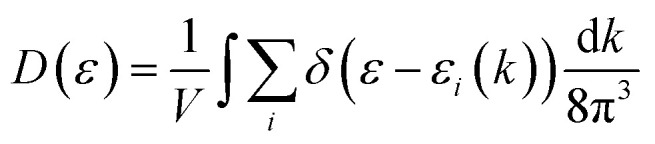
 is the electron density of states, and 
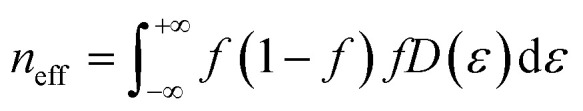
 is the effective carrier density.

In this study, the electrical conductivity, and thus the power factor, is estimated as the relative electrical conductivity *σ*/*τ* (*τ* is the relaxation time) and relative power factor, PF = *σ*^2^*S*/*τ*. The electron relaxation time *τ* cannot be calculated directly in our current work and is usually approximated by a typical value *τ* = 10^−14^ s,^45^ or obtained from fitting the experimental data. Such experimental data is not available yet for the γ-phase and its monolayer. For the calculation of transport properties, we employed a dense *k*-point sampling of 24 × 24 × 1.

## 3 Results and discussion

### 3.1 Crystal structure of monolayer and bulk phases of γ-SnS and γ-SnSe

**Fig. 1:**
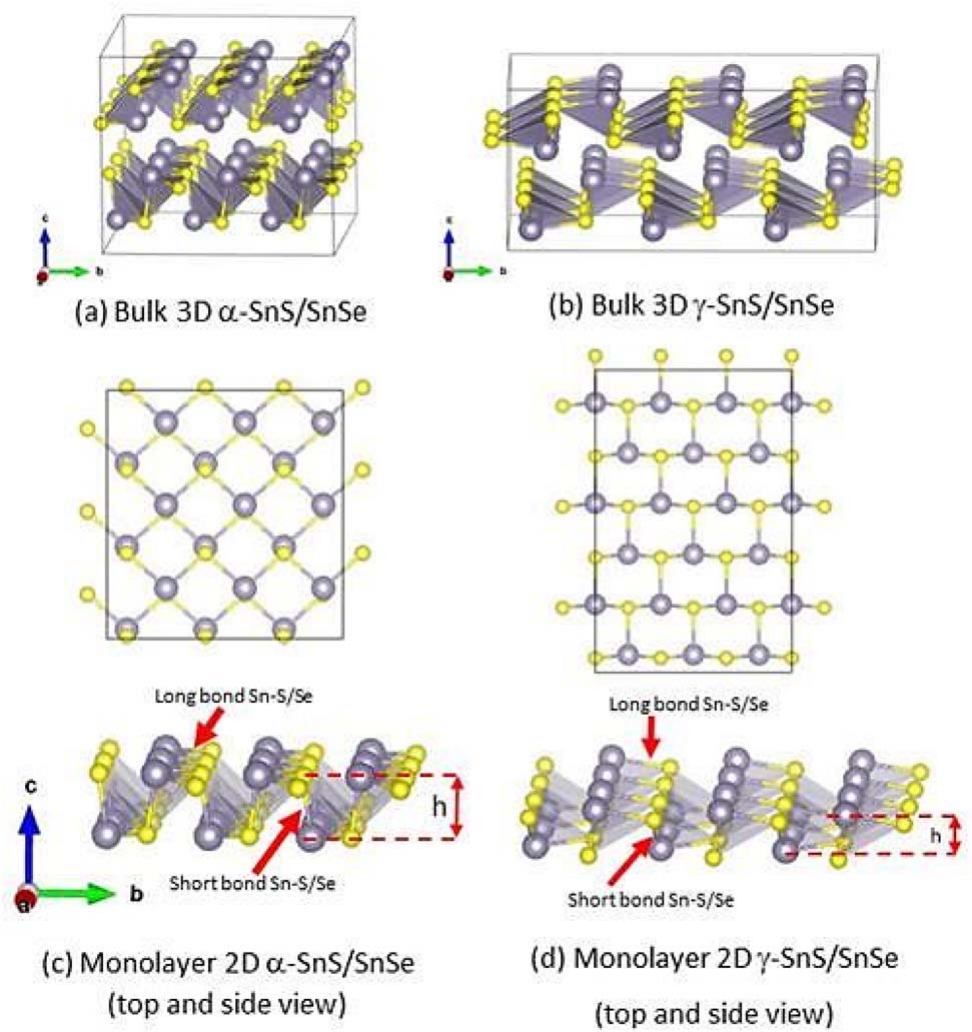
Structures of 3D bulk and 2D monolayers of α- and γ-SnS and SnSe (blue: Sn and yellow: S or Se). (a) Bulk 3D α-structure of SnS and SnSe. (b) Bulk 3D γ-structure of SnS and SnSe. (c) Monolayer 2D α-structure of SnS and SnSe (top and side view). (d) Monolayer 2D γ-structure of SnS and SnSe (top and side view).

The lattice parameters and bond lengths of 2D and 3D γ-SnS and γ-SnSe, compared to their conventional 3D α-*Pnma* counterparts, were calculated and summarized in Table 1. The 2D monolayers of α- and γ-phase of SnS and SnSe are arranged in different hierarchies of the same space group *Pmn*2_1_ (*Z* = 31), as illustrated in Fig. 1. The basic structure of all four phases of SnS and SnSe is a tetrahedron comprising one Sn atom bonded to three chalcogen atoms. While the atomic arrangement of conventional α-SnS and SnSe resemble black phosphorus, γ-SnS and γ-SnSe present a relatively small out-of-plane atomic distortion. We found that lattice parameters of 2D structures of SnS and SnSe are close to their bulk values, within a maximum difference of 0.25 Å.

**Table 1:** The difference in formation energy Δ*E*_f_ (meV per atom) of 3D and 2D α- and γ-SnS and SnSe, 2D surface energy (meV Å^−2^), lattice parameters (Å), thickness *h* (Å), and bond lengths (Å)

Structure	Formation energy Δ*E*_f_ (meV per atom)	2D surface energy (meV Å^−2^)	*a* (Å)	*b* (Å)	*c* (Å)	Layer thickness *h*	Short bond (Å)	Long bond (Å)
Bulk α-SnS	0	—	4.02	4.31	11.26	2.939	2.66	2.69
Bulk γ-SnS	6.46	—	4.01	5.98	8.47	2.216	2.67	2.70
2D α-SnS	147.9	17.6	4.08	4.31	—	2.846	2.59	2.74
2D γ-SnS	158.4	14.8	3.80	5.87	—	2.199	2.61	2.76
Bulk α-SnSe	0	—	4.21	4.56	11.78	3.034	2.78	2.83
Bulk γ-SnSe	2.99	—	4.19	6.23	8.56	2.328	2.80	2.84
2D α-SnSe	165.5	17.9	4.28	4.41	—	2.756	2.73	2.90
2D γ-SnSe	197.6	16.8	3.94	6.19	—	2.002	2.74	2.94

In more detail, the bonding of Sn with chalcogen atoms in a single layer can be classified into two bond types, denoted as the short and long bonds, as shown in Fig. 1c and d. The Sn–S and Sn–Se bond lengths in the α- and γ-structures show slight variations. In the γ-phase, the longer bonds are relatively larger than those in the α-phase, in both 2D and 3D lattices (see Fig. 1). Sn–Se bonds are longer than Sn–S in all cases. The short bond in the 2D structure is shortened while the long bond is elongated compared to their 3D counterparts (see Table 1). The stronger short bond in the 2D structure indicates the increased out-of-plane interaction with respect to the 2D/layer plane, and the stretched long bond represents the weaker in-plane interaction, as seen in Fig. 1. To characterize the distortion of a single layer in both 3D and 2D structures of α- and γ-phases, we calculate its thickness, *h*, as the separation between two layers of Sn atoms, as shown in Fig. 1. We find that the thickness *h* of both 2D monolayers is smaller than the layer thickness in their bulk counterparts.

To compare the energetic stability of 2D and 3D layered structures, we calculated the relative formation energy difference per atom, Δ*E*_f_, for all considered phases of SnS and SnSe with respect to the ground states of bulk α-SnS and α-SnSe as reference values. The equation of the formation energy differences Δ*E*_f_ is expressed as follows:4
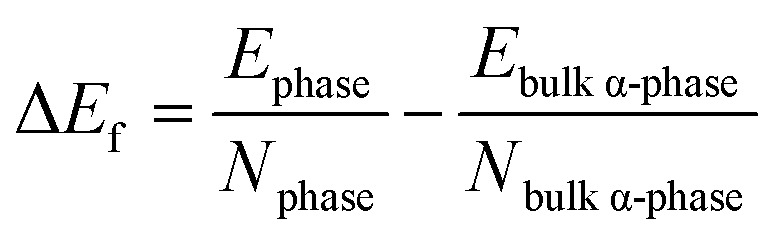
where *E*_phase_ is the total energy of a given phase and *E*_bulk α-phase_ the total energy of the ground states of bulk α-SnS and α-SnSe, normalized by the number of atoms of the corresponding phase. The formation energies of ground states of bulk α-SnS and α-SnSe are referenced to zero eV to determine the difference in energy of the other 3D and 2D phases relative to their corresponding ground states. Our results confirmed that the difference in formation energies between the 3D α- and γ-structures of SnS and SnSe are very small, *i.e.*, several meV, similar to previous work.^13^ This minute difference in formation energy has been validated as bulk γ-SnSe can be obtained through chemical epitaxy on PbS/GaAs substrates in suitable conditions,^18^ and the similar bulk γ-GeSe was synthesized at 6 GPa and 1200 °C, and then remained stable at ambient conditions.^49^ In contrast, the Δ*E*_f_ between 2D α- and γ-structures with respect to the ground-state α-bulk are much higher, from 147.9 meV to 165.5 meV in α-phase and from 158.4 meV to 197.6 meV in γ-phase, see Table 1. Calculated formation energies Δ*E*_f_ with respect to the ground states determine the hull distance of each compound, based on the thermodynamic stability criterion of hull distance proposed from the previous review studies.^50,51^ In general, the energetic stability of metastable 3D or 2D phases can be determined *via* examination of their distance (in energy) above the convex hull of the ground-state phases. Based on existing 2D materials, ref. 50 indicates that only 2D materials with hull distances <200 meV per atom could be synthesized as free-standing monolayers. The hull distance is equivalent to the defined formation energy Δ*E*_f_ relative to the ground-state in eqn (4). From Table 1, 2D γ-phases are less stable than 2D α-phases as they possess a greater hull distance with respect to their ground state. Moreover, 2D SnS is energetically preferred over SnSe in both α- and γ-phases. Compared to the stability criterion for 2D materials of hull energy below 200 meV per atom suggested in ref. 50 2D α- and γ-structures of SnS and SnSe are below this threshold for thermodynamic stability. Furthermore, we calculated another stability criterion of 2D structure to achieve low surface energy.^50^ The surface energy of a 2D material is defined as the unit-cell energy of a 2D material relative to the energy of the lowest energy bulk phase per unit surface area,^50^ shown in the following equation:5
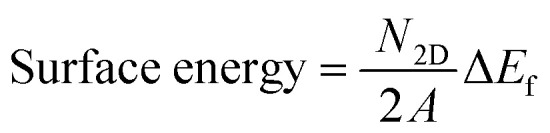
where Δ*E*_f_ is the formation energy relative to the bulk α-ground state, *N*_2D_ is the number of atoms in the 2D cell, and *A* is the surface area. As reported in Table 1 for 2D structures, surface energies of 2D α- and γ-structures of SnS and SnSe satisfy the threshold value of 20 meV Å^−2^.^50^ Therefore, free-standing monolayers of 2D α- and γ-structures of SnS and SnSe are expected to be thermodynamically stable upon synthesis and growth.

**Fig. 2:**
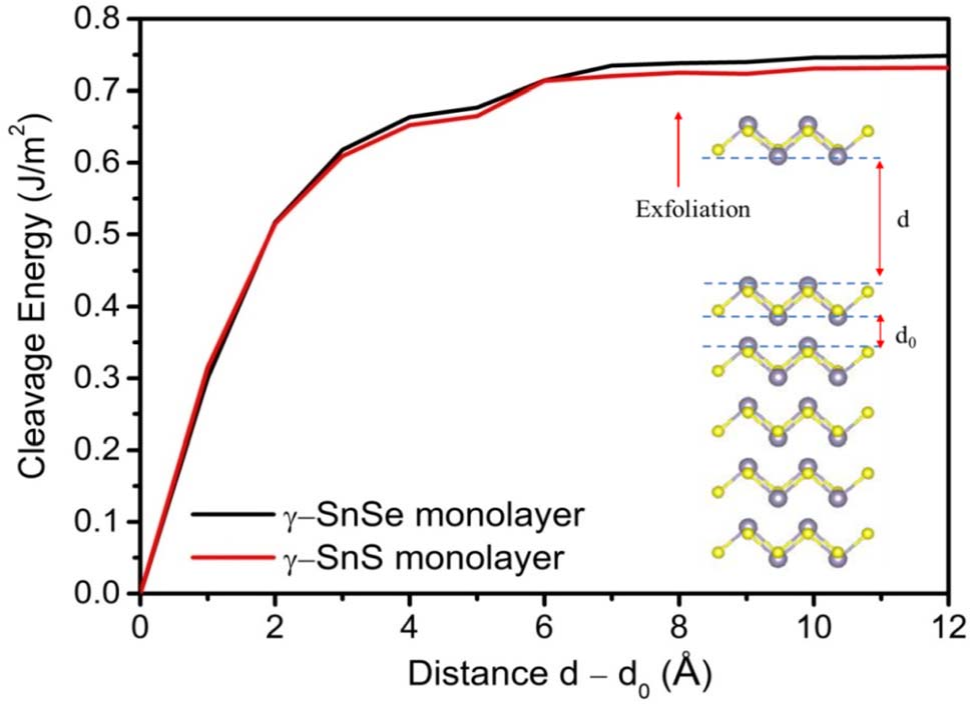
Cleavage energy in J m^−2^ for mechanical exfoliation of γ-structures of SnS and SnSe from 3D bulk phase.

To further evaluate the possible synthesis of 2D phases of γ-SnS and γ-SnSe, we estimate the feasibility of the mechanical exfoliation process to obtain a monolayer structure. Theoretically, the mechanical exfoliation must overcome cleavage energy determined by the interlayer coupling strength between two layers. The cleavage energy can be estimated by employing the extraction of one layer from a supercell containing several layers, as shown in Fig. 2, separated by distance *d* with respect to the interlayer separation *d*_0_ in the bulk configuration, considered an efficient method for obtaining monolayer 2D materials.^27,33,52,53^ Therefore, we evaluate the cleavage energy required to extract one layer of γ-SnS and γ-SnSe from the bulk phase. The cleavage energy presents a gradual increment with the difference distance (*d* − *d*_0_) and converges to a fixed value at approximately 8 Å. Our calculated cleavage energies by mechanical exfoliation of 2D γ-SnS and γ-SnSe converge to values of 0.69 J m^−2^ and 0.71 J m^−2^, respectively, comparable to previous calculations of similar monolayer SnSe,^33,52^ noting that ref. 52 denoted our structure as β-SnSe. The cleavage energy of 2D γ-SnSe is slightly lower than 2D γ-SnS, as seen in Fig. 2. Both cleavage energies of 2D γ-SnS and γ-SnSe are higher than those of pure elemental monolayers such as graphene (0.32–0.37 J m^−2^), phosphorene (0.37 J m^−2^),^25,31,50^ and only slightly higher than the cleavage energies of 2D α-SnSe (0.48 J m^−2^)^27^ and α-GeS (0.52 J m^−2^).^53^ These cleavage energies of γ-SnS and γ-SnSe are considerably lower than in other 2D materials, as GeP_3_ (0.91–1.14 J m^−2^), Ga_2_N (1.09 J m^−2^), and InP_3_ (1.32 J m^−2^).^33,52^ Our results for the surface energy and cleavage energy suggest that the exfoliation of 2D γ-SnS and γ-SnSe from 3D bulk is energetically feasible. However, the high cleavage energy for 2D γ-SnS and γ-SnSe at 0.69 J m^−2^ and 0.71 J m^−2^ predicts difficulty for mechanical exfoliation. We suggest using alternative methods, such as liquid-phase exfoliation or chemical intercalation, to synthesize 2D γ-SnS and γ-SnSe. The close value of cleavage energy hints that similar exfoliation techniques of 2D α-phase could be applied to the recently synthesized bulk phase of γ-SnSe.^19^

**Fig. 3:**
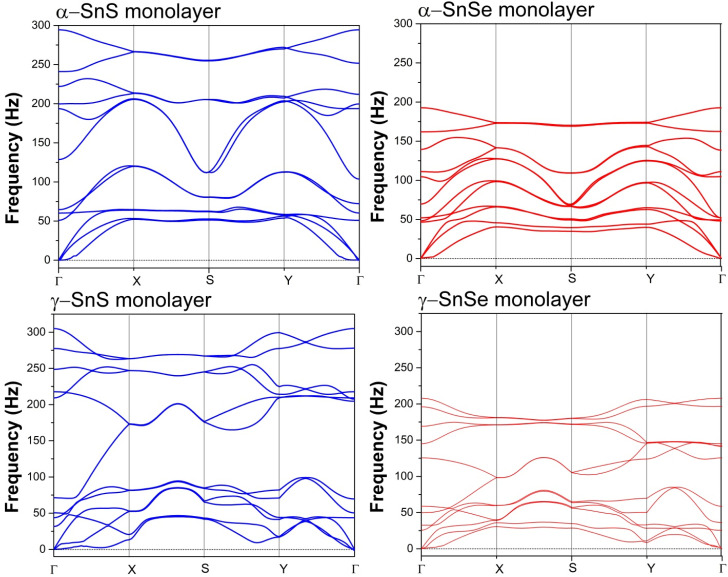
Phonon dispersion curves of 2D α and γ monolayers of SnS and SnSe.

We calculated the phonon dispersion relations for 2D α- and γ-structures of SnS and SnSe as shown in Fig. 3. The positive frequencies obtained across the reciprocal space confirm the dynamical stability of these structures. The phonon spectra for the 2D structures contain a new parabolic branch near the Gamma point, typical of 2D structures, that replaces the third acoustic branch in the 3D structure. Phonon dispersions of 2D SnS and SnSe do not express a phononic band gap, in contrast to the bulk phases of SnSe and SnS, which possess phononic band gaps of 0.7–2 THz in the optical phonon spectrum.^13^Fig. 3 illustrates that the optical phononic frequencies of 2D SnSe are lower than 2D SnS in both monolayer allotropes. This point is interesting as the absence of phononic band gap in our 2D structures is probably preferred for thermoelectric materials. The phononic band gap could result in higher thermal conductivity due to the longer mean free path of acoustic-optical phonon scattering.^54–56^ Bulk α-SnSe possesses ultralow lattice thermal conductivity at around 0.64 W m^−1^ K^−1^ at 300 K and decreases to 0.45 W m^−1^ K^−1^ at 973 K, which is highly desirable in the search for high thermoelectric performance.^14,15^ In addition, materials without a phononic band gap and attaining rattling modes in low-energy phonons could also provide low thermal conductivity.^16^ Hence, the phonon calculation hints that our 2D structures are not only dynamically stable, but could also be favorable candidates for thermoelectric applications.

#### 3.1.1 Electronic properties of 2D γ-SnS and γ-SnSe compared to their allotropes

**Fig. 4:**
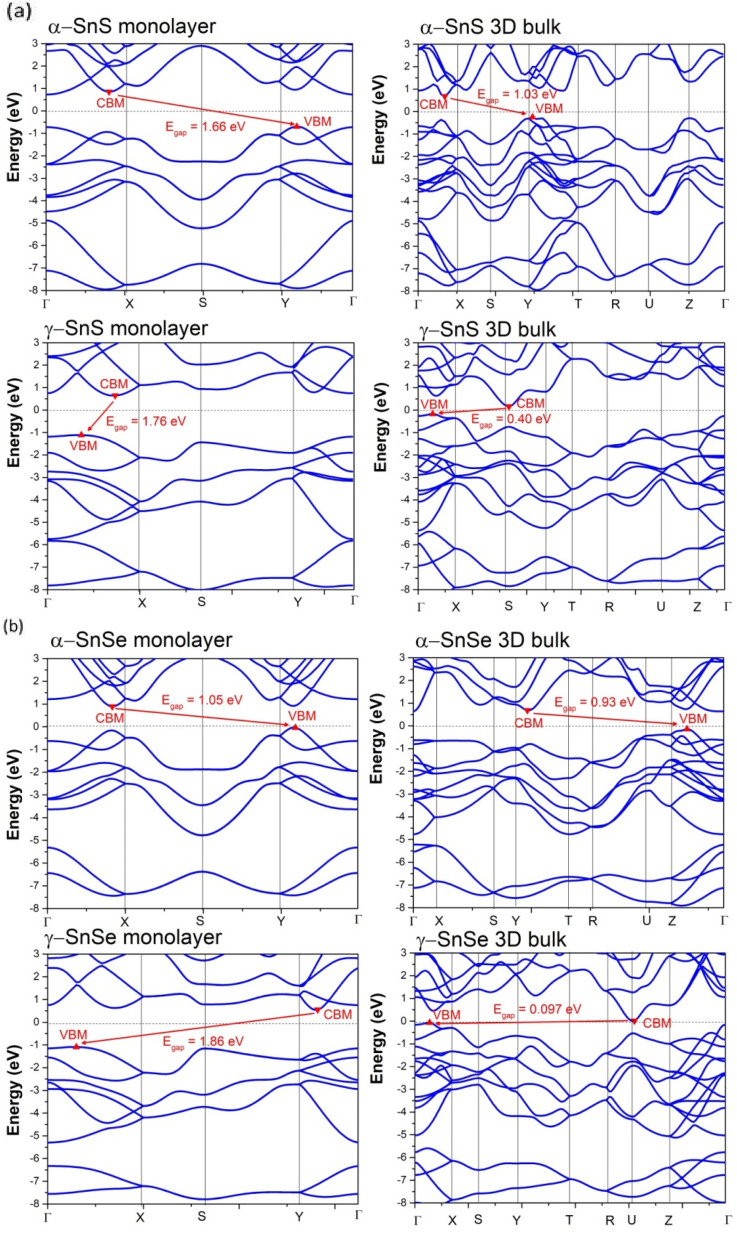
(a) Band structures of α and γ-SnS. (b). Band structures of α and γ-SnSe.

The band structures of α and γ phases of SnS and SnSe were calculated and are presented in Fig. 4a and b, comparable to several previous DFT studies. Our calculated electronic band gaps compared to previous studies are reported in Table 2. Both monolayer and bulk SnS and SnSe are indirect band semiconductors (see Fig. 4). In contrast to bulk SnS and SnSe, 2D monolayers of SnS and SnSe possess wider band gaps. The band gap enlargement of 2D monolayer originates from the lack of interlayer van der Waals interactions and quantum confinement.^61^ Intriguingly, although the band gap enlargement of the 2D α monolayer is considerable relative to the bulk phase, the expansion of the band gap in the 2D γ monolayer is much more dramatic, from 4.4 to 20 times the bulk band gap. Therefore, the band gap may be tuned by controlling the thickness of the layer, *e.g.* bi-layer. We found that 2D γ-monolayers express larger band gaps than 2D α-monolayers while both bulk phases of γ-SnSe and γ-SnS possess narrower band gaps than their α-phase counterparts. For the bulk, the narrower band gap of γ-SnSe (*E*_gap_ = 0.097 eV in bulk γ-SnSe compared to *E*_gap_ = 0.93 eV in bulk α-SnSe) could be driven by the shorter interlayer distance between two layers. Previous combined experimental and theoretical work on bulk γ-SnSe has found that the band gap of γ-SnSe varies monotonically with inter-layer separation while α-SnSe the band gap increases to a fixed value of inter-layer separation and then fluctuate upon increasing to larger inter-layer separation.^19^ Therefore, band gaps in α-phase are essentially independent of the interlayer separation, leading to the band gaps of 2D and bulk α-phase are very close. For γ-phase, the bandgap is very sensitive to the interlayer separation, and thus increase rapidly from 3D to 2D.

**Table 2:** Band gaps (eV) of monolayers and bulk phases of SnS and SnSe

Structure	α-SnS	γ-SnS	Bandgap (eV)	α-SnSe	γ-SnSe
2D monolayer bandgap (eV)	1.66	1.76	2D monolayer	1.05	1.86
	1.83,^61^ 1.56,^30^ 1.58,^57^ 1.82 (ref. 58)	—		1.4,^25^ 0.89,^30^ 0.71,^61^ 0.65,^59^ 0.86 (ref. 24)	1.52,^32^ 2.25 (ref. 33)
CBM	Adjacent X	Between X–Γ	CBM	Adjacent X	Between Y–Γ
VBM	Adjacent Y	Between X–Γ	VBM	Adjacent Y	Between X–Γ
3D bulk bandgap (eV)	1.03	0.40	3D bulk	0.93	0.097
	1.1,^60^ 1.2–1.37 (ref. 58 and 62)	0.45,^13^ 0.59 (ref. 63)		0.89–0.93,^7,58,60^ 1.00 (ref. 62)	0.095 (ref. 19)
CBM	Between X–Γ	S	CBM	Adjacent Y	U
VBM	Y	Between X–Γ	VBM	Adjacent Z	Between X–Γ

**Fig. 5:**
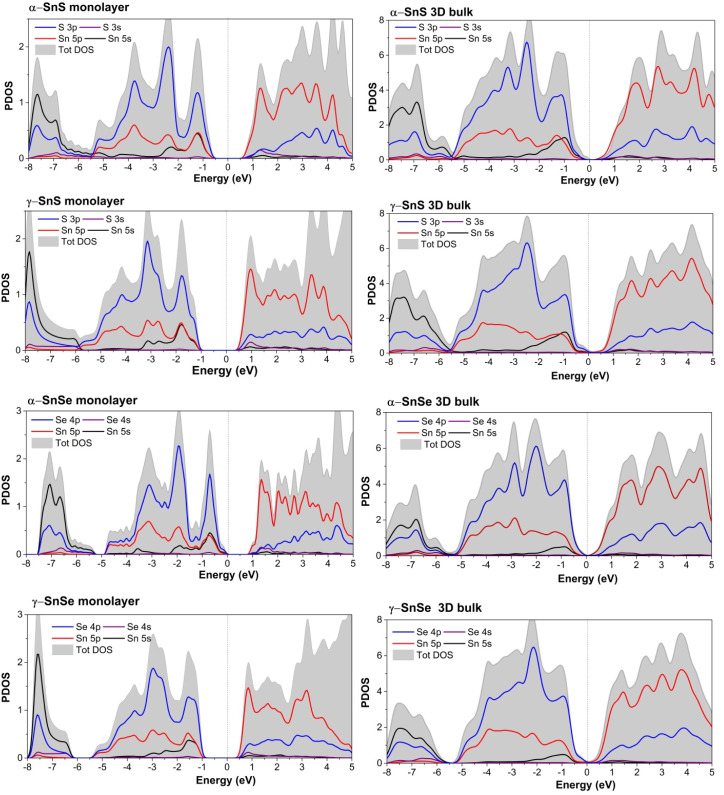
PDOS of 2D monolayer compared to 3D bulk of α and γ structures of SnS and SnSe.

**Fig. 6:**
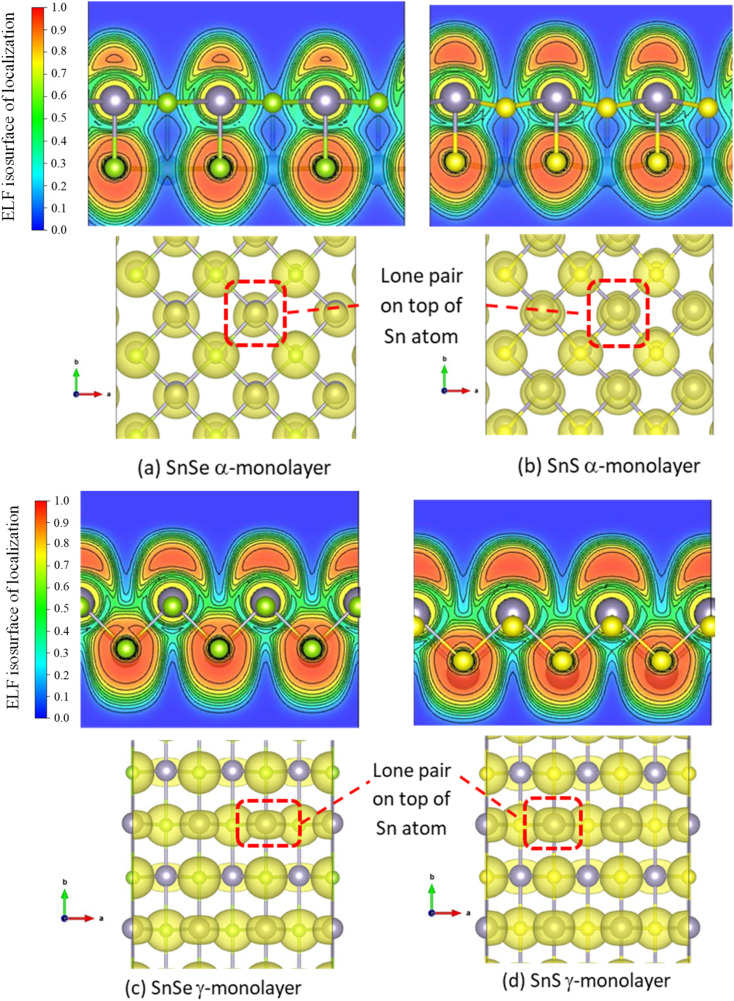
Visualization of lone pairs of 2D and 3D allotropes of α and γ phases of SnS and SnSe by ELF calculation (isosurface at 0.024 eV Å^−3^). We show the 2D display of isosurface section in plane (010) and the top-view of the isosurface of lone pairs. (a) Lone pair from ELF calculation of 2D α-monolayer SnSe. (b) Lone pair from ELF calculation of 2D α-monolayer SnS .(c) Lone pair from ELF calculation of 2D γ-monolayer SnSe. (d) Lone pair from ELF calculation of 2D γ-monolayer SnS.

We calculated the PDOS of α and γ structures in both 3D bulk and 2D monolayer of SnS and SnSe, which are presented in Fig. 5. The overall hybridization of Sn–S and Sn–Se orbitals follows the same scheme in 2D and 3D. The conducting bands just above the Fermi level of α and γ structures are dominated by Sn 5p states with a considerable contribution from chalcogen's p-states. The valence bands below consist mainly of chalcogen's p-states mixed with the Sn 5s and 5p states (see Fig. 5). This hybridization between mixed Sn s–p states with the p-states of the chalcogen in the valence band results in the formation of stereo-active lone pairs.^12,13,64^ A previous study^13^ of metastable phases of SnS and SnSe suggested that the variations in the contributions of the three p-orbitals of chalcogen atoms to the lone-pair led to a wide variety of layered SnS and SnSe structures. In this study, we provide a comparison of 2D and 3D allotropes of α and γ phases of SnS and SnSe (see Fig. 5) and consequently interpret the electronic localized function (ELF) in Fig. 6 to indicate the distinctness of the 2D phase.

**Fig. 7:**
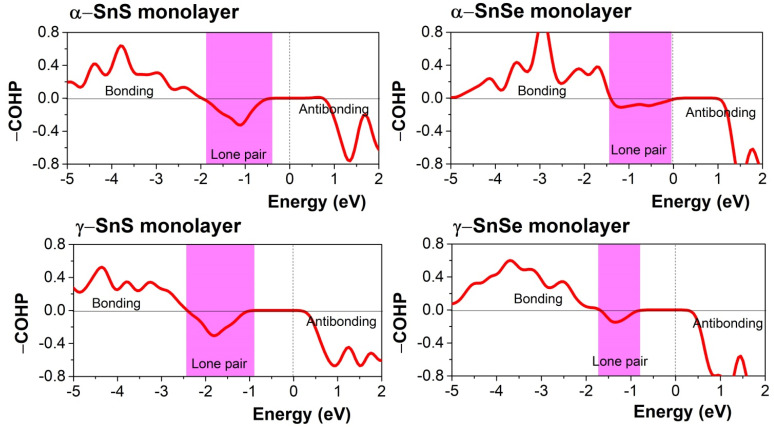
Calculated COHP of 2D monolayers of SnS and SnSe. The energy range of the lone pair is marked by the pink region in COHP, while the antibonding and bonding are displayed by negative and positive of –COHP.

Based on the previous studies of lone pairs in layered SnS and SnSe,^12,13,64^ the localized lone pairs occupy the first peak in the DOS just below the Fermi level, where the Sn 5s orbitals hybridize with Sn 5p and chalcogen's p-orbitals. In Fig. 5, all 2D monolayers exhibit a more pronounced peak in the valence bands below the Fermi level than their respective 3D bulk phases, indicating that the lone pairs are more localized in energy space. Moreover, this first peak in the valence states below the Fermi level also points out another difference between α and γ monolayers, as it is located at a lower energy in the α monolayer than in the γ monolayer. Fig. 6 illustrates the electronic isosurface of the electronic localized function (ELF) for 2D structures of α and γ phases of SnS and SnSe. The localized lone pairs are illustrated by the high-value isosurface, above 0.85 in the color coding, and positioned as a dome above the Sn atom. Fig. 6 shows that lone pairs on the Sn atom in γ-SnSe and γ-SnS occupy larger regions in space than in α monolayers. Another difference between the two 2D allotropes is that the Sn dome lone pairs are generally oriented perpendicular with long bonds, as marked by the red rectangle in Fig. 6. Thus, γ-monolayers possess extended longer bonds than α-monolayers, and this deviation leads to the Sn-dome lone pairs in γ-monolayers being less symmetrical than α-monolayers. Fig. 7 displays the bonding and antibonding states of 2D monolayers *via* COHP calculations. The lone pairs is the antibonding state located below the Fermi level. Herein, the lone pair is primarily formed due to the hybridization of Sn 5s and the p-state of the chalcogen, overlapping with the contribution from the Sn 5p. COHP calculation point out that the lone pairs in γ-monolayers are shifted to lower energy than α-monolayers.

To quantify the difference between the lone pairs of 2D monolayers of SnS and SnSe, we calculated the integrated density of states (IDOS) for the antibonding region of lone pairs, determined by COHP calculation in Fig. 7. These results are represented in Table 3 for a quantitative comparison of the corresponding contributions of each component from Sn and chalcogen states to various hybridizations. The IDOS for lone pairs of γ-monolayers in Table 3 is larger than α-monolayers for all s and p states, confirming that the lone pairs of γ-monolayers are stronger than α-monolayers. IDOS of lone pairs in 2D SnSe is weaker than 2D SnS, which follows the general scheme of lone pairs in this group from ref. 12 and 13 due to the higher energy states of Se 4p than S 3p. We compared the contribution of each p-state in Sn and chalcogen for the 2D case with the reference value of bulk from Table 5 in the previous work.^13^ Interestingly, the contribution of one component in Sn 5p for 2D structures is dominant, while the other two are insignificant. Meanwhile, the contribution from Sn 5p is much weaker in the lone pairs of bulk. For 2D α-SnS and α-SnSe, the strongest contribution is found from the p_*z*_ component while lesser IDOS were provided from the p_*y*_ and p_*x*_ components. Meanwhile, the lone pairs of 2D γ-SnS and γ-SnSe have remarkable contributions from p_*x*_ and p_*z*_ components and one lesser contribution from the p_*y*_ component of S/Se. Bulk α and γ phases in the previous study of the lone pairs feature two motifs.^13^ The first motif of the α-bulk phase has two strong and one weak S/Se p-state contributions. Bulk γ-SnS and γ-SnSe correspond to the hybridization of one strong and two weak contributions from S/Se p-states. Hence, the lone pairs in our 2D structures do not follow the bulk, highlighting the sole contribution of one component of Sn 5p and the distinct contributions of S/Se p-states. Moreover, the stronger lone pairs of γ-monolayers could be the main reason for the remarkable expansion of the band gaps of monolayers compared to their bulks.

**Table 3:** IDOS (number of states) in the lone pairs, integrated in the lone pair's region of the PDOS for each Sn and S/Se contributor. The *z* direction is normal to the monolayer and the *x*- and *y*-directions are in the monolayer

Structure	Sn 5s	Sn total p-states	Sn 5p*_x_*	Sn 5p*_y_*	Sn 5p*_z_*	S/Se total p-states	S/Se p*_x_*	S/Se p*_y_*	S/Se p*_z_*
2D α-SnS	0.278	0.288	0.203	0.029	0.056	0.771	0.216	0.259	0.296
2D α-SnSe	0.239	0.246	0.131	0.048	0.067	0.747	0.227	0.253	0.267
2D γ-SnS	0.307	0.343	0.287	0.022	0.034	1.013	0.394	0.186	0.433
2D γ-SnSe	0.248	0.325	0.248	0.023	0.054	0.948	0.393	0.187	0.368

### 3.2 Electronic transport properties of 2D monolayer for thermoelectric applications

**Fig. 8:**
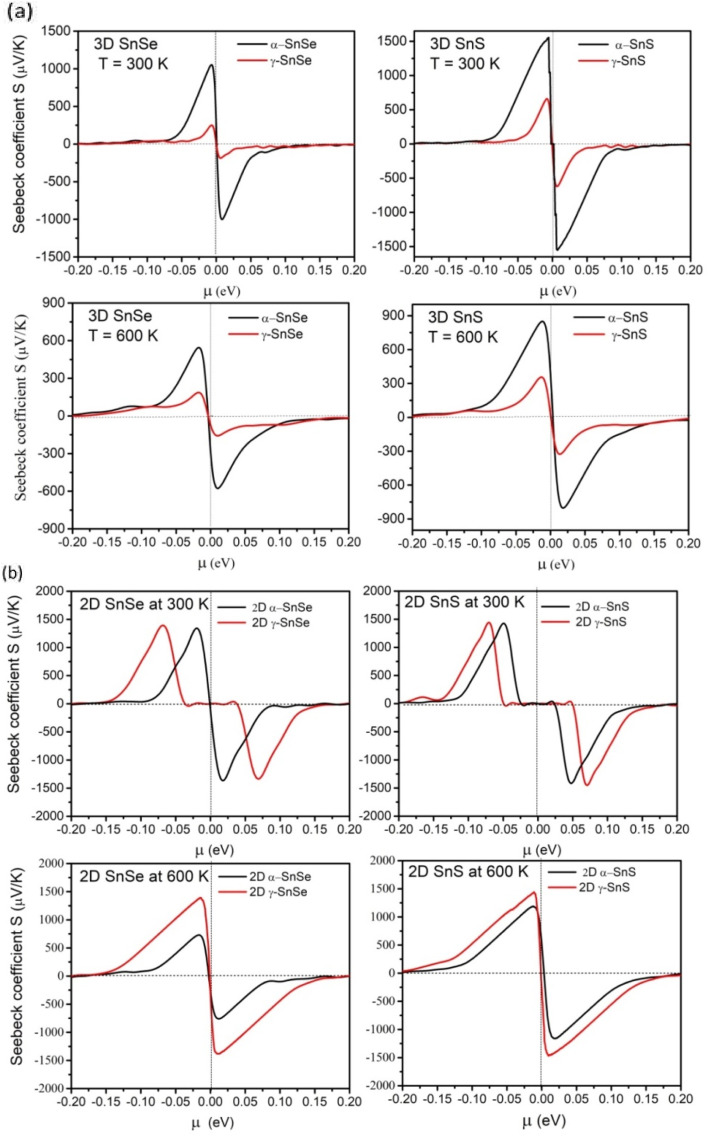
(a) Calculated Seebeck coefficient *S* as a function of chemical potential *μ* (eV) at 300 K and 600 K for bulk 3D α and γ structures of SnS and SnSe. Seebeck coefficient *S* has negative values for n-type carrier *μ* > 0, while for p-type carrier in *μ* < 0, *S* has positive values. (b). Calculated Seebeck coefficient *S* as a function of chemical potential *μ* (eV) at 300 K and 600 K for 2D α and γ structures of SnS and SnSe.

**Fig. 9:**
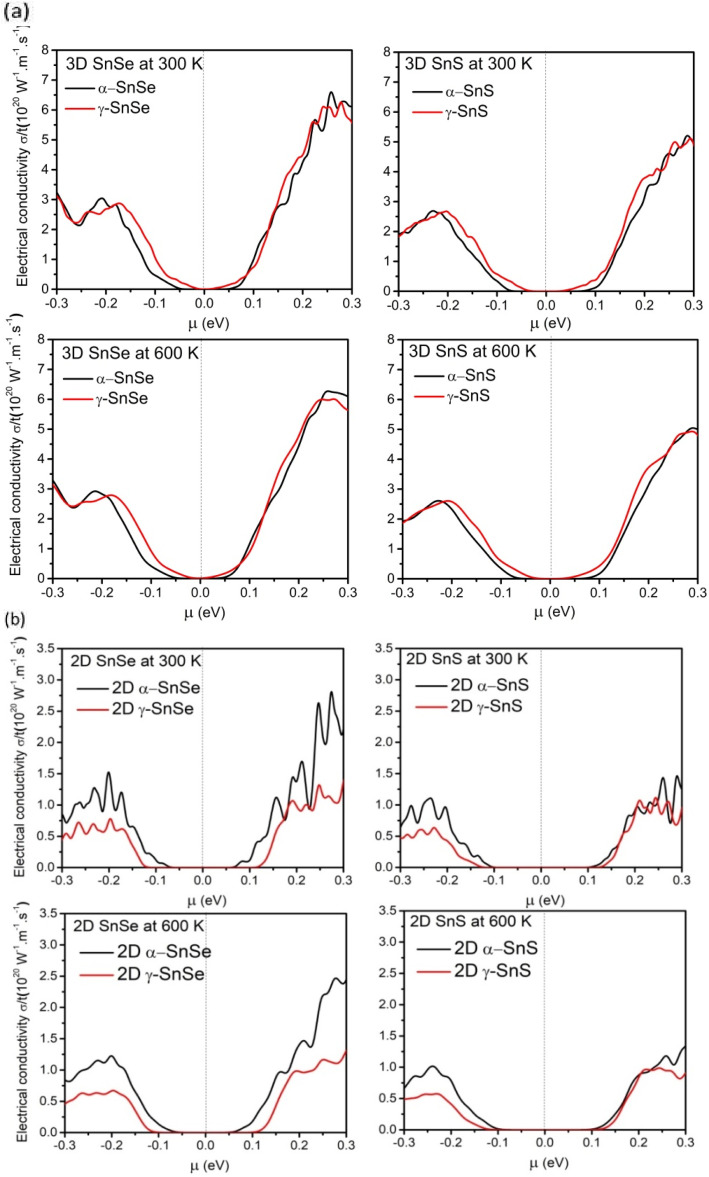
(a). Calculated relative electrical conductivity as a function of chemical potential *μ* (eV) at 300 K and 600 K for 3D α and γ structures of SnS and SnSe. (b). Calculated relative electrical conductivity *σ*/*τ* as a function of chemical potential *μ* (eV) at 300 K and 600 K for 2D α and γ structures of SnS and SnSe.

**Fig. 10:**
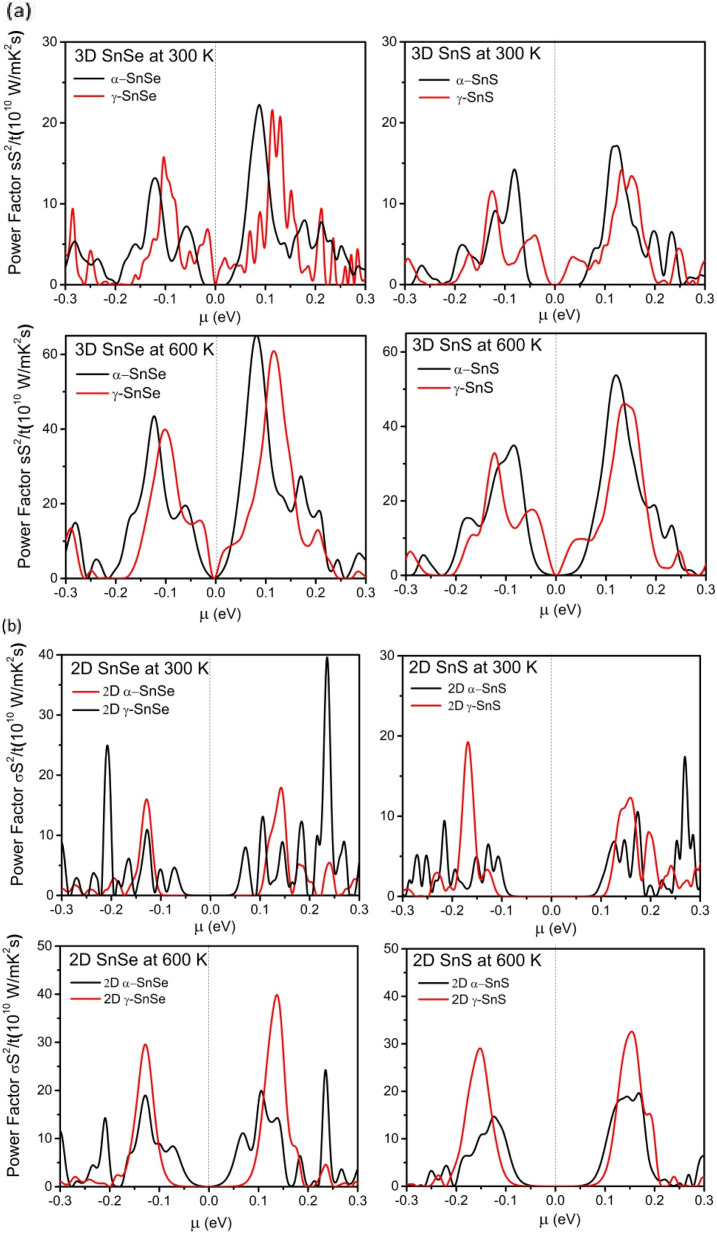
(a) Calculated relative power factor PF as a function of chemical potential *μ* (eV) at 300 K and 600 K for bulk 3D α and γ structures of SnS and SnSe. (b) Calculated relative power factor PF as a function of chemical potential *μ* (eV) at 300 K and 600 K for 2D α and γ structures of SnS and SnSe.

Thermoelectric materials require a large Seebeck coefficient, high electrical conductivity, and low thermal conductivities. To assess the efficiency of function materials in thermoelectric devices these transport properties, and the power factor PF are considered to play an essential role.^57^ Therefore, we calculated them in both 2D and 3D SnS and SnSe to evaluate their efficiency in thermoelectric applications and results are presented in Fig. 8–10. We also compared these electronic transport properties in α and γ structures in 2D and bulk phases.

The Seebeck coefficient of α and γ structures of SnS and SnSe were calculated at 300 K and 600 K in bulk phase (Fig. 8a) and 2D monolayer (Fig. 8b). For bulk α-SnS and α-SnSe, the peak value of Seebeck coefficients are estimated to be 1530 μV K^−1^ (α-SnSe) and 1032 μV K^−1^ (α-SnS) at 300 K and decrease upon heating to 600 K (540 μV K^−1^ for bulk α-SnSe and 850 μV K^−1^ for bulk α-SnS). This trend agrees with previous studies on bulk α-SnSe and α-SnS.^46–48^ Bulk γ-SnSe and γ-SnS show smaller Seebeck coefficients of 279 μV K^−1^ and 679 μV K^−1^ at 300 K that also drop to 186 μV K^−1^ and 355 μV K^−1^ at 600 K, respectively. This trend indicates that the maximum Seebeck coefficients decrease at high temperatures and bulk γ-SnSe/SnS possess overall lower Seebeck coefficients than their α-structure counterparts.^48,65^

For 2D structures in Fig. 8b, the conventional α-monolayers SnS and SnSe exhibit remarkably high peak values of Seebeck coefficients, around 1420–1480 μV K^−1^ at 300 K, and they decrease 730–1120 μV K^−1^ upon heating to 600 K, comparable to the other DFT reports.^46,47^ Hence, the Seebeck coefficient of 2D α-SnSe is higher than its bulk allotrope, while 2D-SnS has nearly the same value as bulk α-SnS. Upon increasing the temperature to 600 K, both Seebeck coefficients of 2D α-SnSe and α-SnS decrease, but to a value higher than in the bulk. Furthermore, peak values of Seebeck coefficients at 300 K for unconventional 2D γ monolayer SnS and SnSe are equal to α-structure, calculated around 1419–1460 μV K^−1^. Intriguingly, upon heating up to 600 K, the peaks of Seebeck coefficients for γ monolayers SnS and SnSe retain high values of 1370 μV K^−1^ for SnSe and 1440 μV K^−1^ for SnS. Thus, Seebeck coefficients of γ monolayers SnS and SnSe can maintain favorable values upon heating to high temperature, which is distinct compared to α-monolayers and bulk phases.

Further evaluating the thermoelectric ability of 2D monolayers of SnS and SnSe, we compared the relative electrical conductivity *σ*/*τ* (*τ* is the relaxation time) at 300 K and 600 K in Fig. 9. In Fig. 9a, we found only a slight difference in relative electrical conductivity between the bulk α and γ structures of SnS and SnSe. For 2D monolayers in Fig. 9b, our results are consistent with other studies as the monolayers have lower electrical conductivity due to their expanded band gap, and all structures exhibit the optimal electrical conductivity in n-type doping, which is generally preferred over the lower peak of electrical conductivity in p-type doping in SnS and SnSe.^22,43,45,55^ Comparing between 2D structures, we found that the electrical conductivity of α monolayers of SnS and SnSe are higher than γ monolayers. In addition, optimal peaks of electrical conductivity of 2D α and γ monolayers SnS are lower than SnSe. For both bulk and 2D monolayers, the overall electrical conductivity of SnS and SnSe decreases slightly upon increased temperature from 300 K to 600 K, consistent with previous findings.^48,65^

To further evaluate the transport properties of thermoelectric devices, we calculate the relative power factor, PF = *σ*^2^*S*/*τ*, and present results for bulk phases in Fig. 10a and for monolayers in Fig. 10b. Similar to previous studies on α-SnSe and α-SnS,^46,48,65^ the optimal power factor of bulk α-SnSe and α-SnS is enhanced upon increased temperature. High power factors of α-SnSe and α-SnS indicate their excellent performance in thermoelectric devices. Furthermore, comparing between bulk α and γ structures of SnS and SnSe, we found that the maximum power factors of γ structures of SnS and SnSe are comparable to their α-bulk allotropes, which could also be increased significantly if working in a high-temperature regime.

For 2D monolayers in Fig. 10b, at 300 K, the α-monolayer of SnSe exhibits the highest peak power factor at *μ* = 0.25, exceeding the maximum power factors of other monolayers of SnS and SnSe. Our result indicates that the optimal power factor of n-type doping is often preferred over p-type in α monolayers and γ monolayer SnSe, similar to the behavior of the bulk in Fig. 10a. An exception is the case of γ monolayers of SnS, which show a higher peak of power factor of p-type doping at 300 K. On the other hand, at 600 K, the maximum power factors of γ-monolayers increased considerably to higher values than that of α-monolayers. These results of optimal power factor suggest that γ monolayers of SnS and SnSe can achieve better values when increase to high temperature. Therefore, both 2D and 3D γ structures of SnS and SnSe have promising potential in thermoelectric applications, along with conventional α-structures.

## 4 Conclusions

In this study, we investigated the crystal structure, dynamical stability, electronic properties, and electronic transport characteristics of bulk and two-dimensional monolayer phases of SnS and SnSe, with a particular focus on the unconventional γ-phase. Our findings reveal that γ-SnS and γ-SnSe monolayers adopt distinct lattice configurations characterized by extended in-plane bonds and higher formation energies compared to their α-phase counterparts. Their surface energy, cleavage energy, and phonon dispersion confirm thermodynamic stability and suggest the feasibility of mechanical exfoliation from the bulk phase. Notably, the calculated cleavage energy for 2D γ-SnS and γ-SnSe at 0.69 J m^−2^ and 0.71 J m^−2^, respectively, predicts that mechanical exfoliation would be difficult and alternative methods such as liquid-phase exfoliation or chemical intercalation are suggested for synthesizing 2D γ-SnS and γ-SnSe.

Electronic structure analysis indicates a significant band gap expansion in 2D γ-SnS and γ-SnSe, particularly in γ-SnSe monolayers. We found that 2D γ-monolayers express larger band gaps than 2D α-monolayers, while both bulk phases of γ-SnSe and γ-SnS possess narrower band gaps than their α-phase counterparts. This variation of the band-gap of 2D γ-SnSe and 2D α-SnSe could be the result of the effect of the lone pairs. Lone-pair interactions are found to be stronger in 2D γ-SnS and γ-SnSe than in their α-phase monolayer counterparts. Additionally, we identify a distinct enhancement of the Sn 5p state contribution and a modified role of the S/Se p-states in the lone-pair formation of the 2D γ-phase compared to the bulk. For the evaluation of electronic transport properties for thermoelectric applications, our theoretical approach provides a comparison of Seebeck coefficients, relative electrical conductivity and power factor as a function of the chemical potential, temperature, and relaxation time. These basic calculations are within the two approximations of the rigid band and constant electron relaxation time. Thus, our study encourages more research into this newly synthesized structure of SnSe and SnS in future work. Comparative assessment of the α- and γ-phases in both bulk and monolayer forms demonstrate that the Seebeck coefficients of γ-SnS and γ-SnSe remain robust at elevated temperatures. Furthermore, the thermoelectric power factors of both bulk and monolayer γ-phases are comparable to or exceed those of the conventional α-phase, underscoring their potential for high-efficiency thermoelectric applications. This work expands our understanding of SnS- and SnSe-based thermoelectric materials. It provides new insights into their structural distortion and electronic versatility, which is promised for next-generation energy conversion technologies.

## Author contributions

Nguyen Truong Long: conceptualization, supervision, methodology, investigation, data curation, writing – original draft preparation, reviewing and editing. Huynh Anh Huy: conceptualization, investigation, writing, reviewing and editing. Neeraj Mishra: methodology, investigation, data curation, writing, reviewing and editing. Guy Makov: conceptualization, investigation, writing, reviewing and editing.

## Data availability

The data that support the findings of this study are available from the corresponding author upon reasonable request.

## Conflicts of interest

There are no conflicts to declare.

## Supplementary Material

RA-015-D5RA01965F-s001
