# Supplementary material for: Theoretical study of electronic structure, lone pair localization, and electronic transport properties of unconventional bulk and 2D γ-SnSe and γ-SnS
Source: RSC Adv. 2025 May 16;15(21):16358–74. doi: 10.1039/d5ra01965f (PMC12082441; doi:10.1039/d5ra01965f)
Supplement: RA-015-D5RA01965F-s001 [file RA-015-D5RA01965F-s001.pdf]

Supplementary Information (SI) for RSC Advances.
This journal is © The Royal Society of Chemistry 2025

SUPPLEMENTARY INFORMATION

Theoretical study of electronic and transport thermoelectric properties of unconventional bulk and 2D γ-SnSe and γ-SnS

Nguyen Truong Long[a*], Huynh Anh Huy[a], Neeraj Mishra[b], Guy Makov[b]

[a.] *School of Physics Education, Can Tho University, Can Tho 900000, Viet Nam*
[b.] *Dept. of Materials Engineering, Ben-Gurion University of the Negev, Beer Sheva 84105, Israel*

*E-mail: truonglong@ctu.edu.vn

Structure data in CIF format for 2D and bulk SnS and SnSe phases

```
#======================================================================
=
# CRYSTAL DATA
#----------------------------------------------------------------------
-
data_SnSe_3D_alpha

_chemical_name_common                  'SnSe'
_cell_length_a                         4.211737
_cell_length_b                         4.557569
_cell_length_c                         11.782299
_cell_angle_alpha                      90.000000
_cell_angle_beta                       90.000000
_cell_angle_gamma                      90.000000
_cell_volume                           226.164562
_space_group_name_H-M_alt              'P 1'
_space_group_IT_number                 1

loop_
_space_group_symop_operation_xyz
   'x, y, z'

loop_
   _atom_site_label
   _atom_site_occupancy
   _atom_site_fract_x
   _atom_site_fract_y
   _atom_site_fract_z
   _atom_site_adp_type
   _atom_site_U_iso_or_equiv
   _atom_site_type_symbol
   Sn1        1.0     0.249999     0.383412     0.621252    Uiso  ? Sn
```

```
   Sn2        1.0     0.249999     0.116589     0.121252    Uiso  ? Sn
   Sn3        1.0     0.750000     0.616587     0.378748    Uiso  ? Sn
   Sn4        1.0     0.750000     0.883413     0.878748    Uiso  ? Sn
   Se1        1.0     0.249999     0.025452     0.355290    Uiso  ? Se
   Se2        1.0     0.249999     0.474549     0.855290    Uiso  ? Se
   Se3        1.0     0.750000     0.974547     0.644710    Uiso  ? Se
   Se4        1.0     0.750000     0.525453     0.144710    Uiso  ? Se


#======================================================================
=
# CRYSTAL DATA
#----------------------------------------------------------------------
-
data_SnSe_2D_alpha

_chemical_name_common                  'SnSe'
_cell_length_a                         4.283233
_cell_length_b                         4.410285
_cell_length_c                         25.585487
_cell_angle_alpha                      90.000000
_cell_angle_beta                       90.000000
_cell_angle_gamma                      90.000000
_cell_volume                           483.316994
_space_group_name_H-M_alt              'P 1'
_space_group_IT_number                 1

loop_
_space_group_symop_operation_xyz
   'x, y, z'

loop_
   _atom_site_label
   _atom_site_occupancy
   _atom_site_fract_x
   _atom_site_fract_y
   _atom_site_fract_z
   _atom_site_adp_type
   _atom_site_U_iso_or_equiv
   _atom_site_type_symbol
   Sn1        1.0     0.750000     0.600613     0.160825    Uiso  ? Sn
   Sn2        1.0     0.250000     0.101418     0.053099    Uiso  ? Sn
   Se1        1.0     0.250000     0.045498     0.159362    Uiso  ? Se
   Se2        1.0     0.750000     0.545867     0.054547    Uiso  ? Se


#======================================================================
=
# CRYSTAL DATA
#----------------------------------------------------------------------
-
data_SnSe_3D_gamma
```

```
_chemical_name_common                  'SnSe'
_cell_length_a                         4.192973
_cell_length_b                         8.557502
_cell_length_c                         6.232721
_cell_angle_alpha                      90.029701
_cell_angle_beta                       90.012184
_cell_angle_gamma                      89.986389
_cell_volume                           223.638554
_space_group_name_H-M_alt              'P 1'
_space_group_IT_number                 1

loop_
_space_group_symop_operation_xyz
   'x, y, z'

loop_
   _atom_site_label
   _atom_site_occupancy
   _atom_site_fract_x
   _atom_site_fract_y
   _atom_site_fract_z
   _atom_site_adp_type
   _atom_site_U_iso_or_equiv
   _atom_site_type_symbol
   Sn1        1.0     0.627416     0.603214     0.700032    Uiso  ? Sn
   Sn2        1.0     0.126418     0.874972     0.199680    Uiso  ? Sn
   Sn3        1.0     0.628084     0.103166     0.822760    Uiso  ? Sn
   Sn4        1.0     0.126022     0.375046     0.323304    Uiso  ? Sn
   Se1        1.0     0.127396     0.823296     0.643752    Uiso  ? Se
   Se2        1.0     0.626024     0.654786     0.143914    Uiso  ? Se
   Se3        1.0     0.626784     0.154854     0.378654    Uiso  ? Se
   Se4        1.0     0.127348     0.323350     0.879080    Uiso  ? Se



#======================================================================
=
# CRYSTAL DATA
#----------------------------------------------------------------------
-
data_SnSe_2D_gamma

_chemical_name_common                  'SnSe'
_cell_length_a                         3.937749
_cell_length_b                         6.186510
_cell_length_c                         23.581026
_cell_angle_alpha                      90.000000
_cell_angle_beta                       90.000000
_cell_angle_gamma                      90.000000
_cell_volume                           574.455569
```

```
_space_group_name_H-M_alt              'P 1'
_space_group_IT_number                 1

loop_
_space_group_symop_operation_xyz
   'x, y, z'

loop_
   _atom_site_label
   _atom_site_occupancy
   _atom_site_fract_x
   _atom_site_fract_y
   _atom_site_fract_z
   _atom_site_adp_type
   _atom_site_U_iso_or_equiv
   _atom_site_type_symbol
   Sn1        1.0     0.500000     0.172354     0.048531    Uiso  ? Sn
   Sn2        1.0     0.000000     0.672516     0.133419    Uiso  ? Sn
   Se1        1.0     0.000000     0.147411     0.128777    Uiso  ? Se
   Se2        1.0     0.500000     0.647450     0.053168    Uiso  ? Se




#======================================================================
=
# CRYSTAL DATA
#----------------------------------------------------------------------
-
data_SnS_3D_alpha

_chemical_name_common                  'SnSe'
_cell_length_a                         11.258350
_cell_length_b                         4.020000
_cell_length_c                         4.318500
_cell_angle_alpha                      90.000000
_cell_angle_beta                       90.000000
_cell_angle_gamma                      90.000000
_cell_volume                           195.449129
_space_group_name_H-M_alt              'P n m a'
_space_group_IT_number                 62

loop_
_space_group_symop_operation_xyz
   'x, y, z'
   '-x, -y, -z'
   '-x+1/2, -y, z+1/2'
   'x+1/2, y, -z+1/2'
   '-x, y+1/2, -z'
   'x, -y+1/2, z'
```

```
   'x+1/2, -y+1/2, -z+1/2'
   '-x+1/2, y+1/2, z+1/2'

loop_
   _atom_site_label
   _atom_site_occupancy
   _atom_site_fract_x
   _atom_site_fract_y
   _atom_site_fract_z
   _atom_site_adp_type
   _atom_site_B_iso_or_equiv
   _atom_site_type_symbol
   Sn1        1.0     0.619470     0.250000     0.380870    Biso
1.000000 Sn
   S1         1.0     0.352700     0.250000     0.027510    Biso
1.000000 S



#======================================================================
=
# CRYSTAL DATA
#----------------------------------------------------------------------
-
data_SnS_2D_alpha

_chemical_name_common                  'SnS'
_cell_length_a                         4.078460
_cell_length_b                         4.310366
_cell_length_c                         25.585487
_cell_angle_alpha                      90.000000
_cell_angle_beta                       90.000000
_cell_angle_gamma                      90.000000
_cell_volume                           449.784089
_space_group_name_H-M_alt              'P 1'
_space_group_IT_number                 1

loop_
_space_group_symop_operation_xyz
   'x, y, z'

loop_
   _atom_site_label
   _atom_site_occupancy
   _atom_site_fract_x
   _atom_site_fract_y
   _atom_site_fract_z
   _atom_site_adp_type
   _atom_site_U_iso_or_equiv
   _atom_site_type_symbol
   Sn1        1.0     0.750000     0.613005     0.162589    Uiso  ? Sn
```

```
   Sn2        1.0     0.249999     0.113157     0.051357    Uiso  ? Sn
   S1         1.0     0.249999     0.033921     0.151908    Uiso  ? S
   S2         1.0     0.750000     0.533310     0.061979    Uiso  ? S



#======================================================================
=
# CRYSTAL DATA
#----------------------------------------------------------------------
-
data_SnS_3D_gamma

_chemical_name_common                  'SnS'
_cell_length_a                          4.018080
_cell_length_b                          8.471977
_cell_length_c                          5.977116
_cell_angle_alpha                       90.000000
_cell_angle_beta                        90.000000
_cell_angle_gamma                       90.000000
_cell_volume                            203.467513
_space_group_name_H-M_alt              'P 1'
_space_group_IT_number                  1

loop_
_space_group_symop_operation_xyz
   'x, y, z'

loop_
   _atom_site_label
   _atom_site_occupancy
   _atom_site_fract_x
   _atom_site_fract_y
   _atom_site_fract_z
   _atom_site_adp_type
   _atom_site_B_iso_or_equiv
   _atom_site_type_symbol
   Sn1        1.0     0.500010     0.380760     0.187344    Biso
1.000000 Sn
   Sn2        1.0     0.000010     0.619240     0.812654    Biso
1.000000 Sn
   Sn3        1.0     0.999990     0.119240     0.687346    Biso
1.000000 Sn
   Sn4        1.0     0.499990     0.880760     0.312656    Biso
1.000000 Sn
   S1         1.0     0.000002     0.672224     0.371972    Biso
1.000000 S
   S2         1.0     0.500002     0.327776     0.628028    Biso
1.000000 S
   S3         1.0     0.499998     0.827776     0.871972    Biso
1.000000 S
```

```
   S4         1.0     0.999998     0.172224     0.128028    Biso
1.000000 S




#======================================================================
=
# CRYSTAL DATA
#----------------------------------------------------------------------
-
data_SnS_2D_gamma

_chemical_name_common                    'SnS'
_cell_length_a                           3.803752
_cell_length_b                           5.866401
_cell_length_c                           23.581026
_cell_angle_alpha                        90.000000
_cell_angle_beta                         90.000000
_cell_angle_gamma                        90.000000
_cell_volume                             526.194915
_space_group_name_H-M_alt                'P 1'
_space_group_IT_number                   1

loop_
_space_group_symop_operation_xyz
   'x, y, z'

loop_
   _atom_site_label
   _atom_site_occupancy
   _atom_site_fract_x
   _atom_site_fract_y
   _atom_site_fract_z
   _atom_site_adp_type
   _atom_site_U_iso_or_equiv
   _atom_site_type_symbol
   Sn1        1.0     0.500001     0.177075     0.044359    Uiso  ? Sn
   Sn2        1.0     0.000000     0.677037     0.137590    Uiso  ? Sn
   S1         1.0     0.000000     0.142821     0.119766    Uiso  ? S
   S2         1.0     0.500001     0.642798     0.062180    Uiso  ? S
```
